# Identification of superior jujube (*Ziziphus jujuba* Mill.) genotypes based on morphological and fruit characterizations

**DOI:** 10.1002/fsn3.2276

**Published:** 2021-04-05

**Authors:** Ali Khadivi, Farhad Mirheidari, Younes Moradi, Simin Paryan

**Affiliations:** ^1^ Department of Horticultural Sciences Faculty of Agriculture and Natural Resources Arak University Arak Iran

**Keywords:** breeding, conservation, fruit, jujube, medicinal properties

## Abstract

Jujube (*Ziziphus jujuba* Mill.) is of great importance due to its medicinal properties and nutritional value. The current investigation was carried out to assess phenotypic variation of naturally grown accessions of this species. The accessions investigated exhibited meaningful variabilities based on the recorded characters. Fruit fresh weight ranged from 0.36 to 3.83 g with an average of 1.16, fruit dry weight varied from 0.21 to 3.04 g with an average of 0.80, and fruit flesh thickness varied from 1.24 to 8.51 mm. Skin color showed large variabilities among the accessions, including maroon‐yellow, light maroon, maroon, dark maroon, light brown, brown, dark brown, and maroon‐crimson. Principal component analysis (PCA) determined the characters influencing the most variation among the accessions. Cluster analysis performed with the Euclidean distance and Ward's method divided the accessions into two main clusters. The present findings provided essential data about the morphological traits of *Z. jujube* that can be used for the selection of superior genotypes and development of the fruit processing industry. Besides, the available results can be useful in designing conservation strategies and breeding of *Z. jujube*.

## Graphical abstract

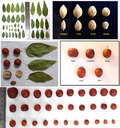

## 1 INTRODUCTION

Jujube (*Ziziphus jujuba* Mill., the Rhamnaceae family), originated from China, is of great importance due to its nutritional value and medicinal properties (Reche et al., 2018). This plant species is distributed in mild‐temperate to subtropical regions and is naturally adapted to areas with cool winters and hot summers. Cultivars and varieties have a wide variety of traits such as fruit shape, taste, and color (Ma et al., 2011).

Tolerance of jujube to some environmental stresses such as drought, salinity, and some pests and diseases is high. Its fruit is organic because of its production by applying less pesticide (Velkoska‐Markovska & Petanovska‐Ilievska, 2013). Easy harvesting, high yield and price, high adaptability range, easy management, early bearing, and high nutritional value are the unique characteristics of jujube.

The fruit of *Z. jujuba* has a high nutritional value and is rich in vitamin C and amino acids (Wang et al., 2011). Other valuable nutrients in this fruit include carbohydrates, potassium, iron, and phenolic compounds. Different parts of jujube have multiple medicinal properties. Jujube seeds are used for their activity in alleviating insomnia and anxiety (Wang et al., 2011).

Genetic variation in a plant species is the basis of tree breeding programs. Breeding programs should be begun by identifying important traits and performed using diversity in natural populations. In each country, studying the genetic diversity of a plant species is important for use in breeding, conservation, and management (Awasthi & More, 2009). Therefore, identifying native and local genotypes is important to recognize ones with desired fruit traits, environmental adaptation, and resistance to environmental stress. Morphological diversity is the basis of the evolution of plants, and thus the valuation of morphological traits is the first step in determining genetic diversity and classification of plants (Khadivi et al., 2019).

Genetic diversity of most plants, including fruit trees, in Iran is considerable, because the climatic conditions in the country are diverse, ranging from subtropical to temperate. Fruit trees play an important role in the country's economy (Khadivi et al., 2019). Medicinal fruit species are well distributed in the country, and their attention can help breeding and conservation programs (Goodarzi et al., 2018). Therefore, in the present study, the morphological diversity of the native accessions of *Z. jujuba* was studied. This study is one of the first studies in Iran to investigate the morphological characteristics of a large number of accessions of this species that can provide useful information for determining genetic diversity which can be used for conservation and breeding of this plant.

## 2 MATERIALS AND METHODS

### 2.1 Plant material

The current investigation was carried out to assess phenotypic variation of 140 accessions of *Z. jujuba* naturally grown in Markazi (three areas) and Isfahan (six areas) provinces in Iran. The geographical coordinates of the studied collection sites have been shown in Table 1.

**TABLE 1:** Geographical description for the collection sites of *Z. jujuba* accessions investigated

No.	Province	Area	Latitude (*N*)	Longitude (E)	Altitude (m)	Sample size
1	Markazi	Mamoonyeh	35°17′29″	50°31′06″	1,276	38
2	Markazi	Khoshkrood	35°24′31″	50°21′02″	1584	5
3	Markazi	Barbar	35°19′32″	50°29′08″	1,282	3
4	Isfahan	Isfahan	32°36′04″	51°41′35″	1649	27
5	Isfahan	Zardenjan	32°34′02″	51°47′13″	1554	17
6	Isfahan	Khatoonabad	32°39′44″	51°47′12″	1556	20
7	Isfahan	Payekan	32°14′54″	52°10′13″	1593	13
8	Isfahan	Tinejan	32°50′47″	52°26′40″	2,287	8
9	Isfahan	Koohpayeh	32°49′39″	52°26′19″	1757	9

### 2.2 The characters evaluated

In total, 49 morphological characters were used for phenotypic evaluations (Table 2). Seven characters including leaf length, leaf width, petiole length, fruit length, fruit width, seed length, and seed width were measured using a digital caliper. Fruit weight and seed weight were measured using an electronic balance with 0.01 g precision. Besides, 19 qualitative characters were estimated based on rating and coding (Table 2).

**TABLE 2:** Descriptive statistics for morphological traits utilized in the studied *Z. jujuba* accessions

No.	Character	Abbreviation	Unit	Min	Max	Mean	*SD*	CV (%)
1	Tree growth habit	TGH	Code	1	9	6.27	2.27	36.14
2	Tree vigor	TV	Code	1	5	4.03	1.30	32.33
3	Tree height	THe	Code	1	5	3.49	1.50	42.87
4	Branching	B	Code	1	5	3.57	1.47	41.04
5	Branch density	BD	Code	1	5	3.59	1.49	41.45
6	Branch flexibility	BF	Code	1	5	2.47	1.39	56.32
7	Trunk color	TrC	Code	1	11	5.69	3.17	55.75
8	Trunk type	TrTy	Code	1	3	1.46	0.84	57.74
9	Trunk diameter	TrDi	Code	1	5	3.54	1.10	30.93
10	Canopy symmetry	CaSy	Code	0	1	0.68	0.47	68.97
11	Canopy density	CaD	Code	1	5	3.31	1.65	49.82
12	Tendency to produce suckers	TeSu	Code	0	5	0.91	1.10	121.21
13	Leaf density	LD	Code	1	5	4.39	1.10	24.99
14	Leaf shape	LSh	Code	1	5	2.74	1.57	57.30
15	Leaf apex shape	LASh	Code	1	5	2.27	1.60	70.26
16	Leaf length	LLe	mm	26.33	84.05	44.23	9.75	22.05
17	Leaf width	LWi	mm	11.02	32.87	19.23	3.84	19.96
18	Leaf upper surface color	LUSuC	Code	1	5	3.56	0.99	27.84
19	Leaf lower surface color	LLoSuC	Code	1	3	1.47	0.85	57.96
20	Leaf margin serration	LMSe	Code	0	1	0.97	0.17	17.22
21	Leaf serration depth	LSeDe	Code	0	5	2.61	1.54	59.16
22	Leaf serration shape	LSeSh	Code	1	7	3.93	1.53	38.80
23	Petiole length	PeLe	mm	1.55	10.60	4.43	1.98	44.76
24	Petiole thickness	PeWi	mm	0.37	1.39	0.67	0.15	22.99
25	Thorn presence on current shoot	ThoPrCu	Code	0	1	0.06	0.25	410.00
26	Thorn number on annual shoot	ThoNoAn	Number	0	32	10.42	5.84	56.02
27	Thorn length on annual shoot	ThoLeAn	mm	0.00	38.84	10.88	8.76	80.54
28	Thorn base thickness on annual shoot	ThoBaTh	mm	0.00	4.75	1.58	0.99	62.53
29	Ripening time	RiTi	Date	Late Sep	Early Oct	1.23	0.64	51.95
30	Fruit yield	FrYi	Code	1	5	3.59	1.39	38.66
31	Fruit length	FrLe	mm	9.43	49.47	14.22	3.94	27.68
32	Fruit width	FrWi	mm	8.38	30.17	13.28	2.80	21.07
33	Fruit fresh weight	FrFlWe	g	0.36	3.83	1.16	0.56	48.62
34	Fruit dry weight	FrDrWe	g	0.21	3.04	0.80	0.43	53.88
35	Fruit shape	FrSh	Code	1	9	5.23	2.62	50.06
36	Fruit skin transparency	FrSkTr	Code	1	5	2.79	1.37	49.18
37	Fruit stalk length	FrStLe	mm	1.19	8.81	3.24	1.13	34.72
38	Fruit stalk diameter	FrStDi	mm	0.35	2.98	0.83	0.25	29.64
39	Fruit skin color	FrSkC	Code	1	15	8.31	3.36	40.40
40	Fruit flesh color	FrFlC	Code	1	11	6.23	2.91	46.77
41	Fruit flesh thickness	FrFlTh	mm	1.24	8.51	2.81	1.05	37.37
42	Fruit taste	FrTa	Code	1	7	4.59	1.39	30.24
43	Fruit flesh texture	FrFlTe	Code	1	3	1.54	0.89	57.99
44	Stone length	StLe	mm	6.76	15.57	9.75	1.70	17.41
45	Stone width	StWi	mm	3.54	9.05	6.13	1.09	17.80
46	Stone weight	StWe	g	0.04	0.53	0.19	0.09	46.32
47	Stone shape	StSh	Code	1	7	3.07	2.06	66.94
48	Stone surface	StSu	Code	1	5	4.61	0.99	21.39
49	Stone terminal appendix	StApp	Code	0	3	0.56	1.03	184.46

### 2.3 Statistical analysis

Analysis of variance (ANOVA) was performed to evaluate the variation among the accessions based on the traits measured using SAS software (SAS Institute, Cary, NC, & USA, 1990). The Pearson correlation coefficients were used to determine correlations between the characters with SPSS software (SPSS Inc., Chicago, IL, USA, Norusis, 1998). The relationship between the accessions and the main traits effective in accessions segregation was determined using principal component analysis (PCA) with SPSS software. Hierarchical cluster analysis (HCA) was performed using Ward's method and Euclidean coefficient using PAST software (Hammer et al., 2001). Besides, PAST software was applied to generate a scatter plot using the first and second principal components (PC1/PC2).

## 3 RESULTS AND DISCUSSION

### 3.1 Morphological characterizations

The accessions investigated exhibited meaningful variabilities based on the recorded characters (ANOVA, *p* <.01). In general, 45 out of 49 characters measured (91.84% in total) showed the CVs more than 20.00%. The highest CV was related to the thorn presence on current shoot (410.00%) and followed by stone terminal appendix (184.46%), tendency to produce suckers (121.21%), and thorn length on the annual shoot (80.54%), while the lowest CVs belonged to leaf margin serration (17.22%), stone length (17.41%), stone width (17.80%), and leaf width (19.96%) (Table 2), respectively. Tous et al., (1995) suggested that the CV of less than 10.00% is considered low, 10.00 to 20.00% is moderate, and more than 20.00% is considered high in fruit trees.

Tree growth habit was predominantly open (55 accessions). Tree vigor, tree height, branching, branch density, canopy density, and leaf density showed high values and followed by intermediate. The majority of accessions were single‐trunk type (108). Three types of leaf shape were observed, including ovate (53 accessions), lanceolate (52), and elliptical (35) (Table 3).

**TABLE 3:** Frequency distribution of the measured qualitative morphological characters in the studied *Z. jujuba* accessions

Character	Frequency (no. of accessions)						
	0	1	3	5	7	9	11
Tree growth habit	‐	Weeping (8)	Spreading (7)	Open (55)	Semi‐erect (28)	Erect (42)	‐
Tree vigor	‐	Low (12)	Intermediate (44)	High (84)	‐	‐	‐
Tree height	‐	Small (26)	Intermediate (54)	Tall (60)	‐	‐	‐
Branching	‐	Low (23)	Intermediate (54)	High (63)	‐	‐	‐
Branch density	‐	Low (24)	Intermediate (51)	High (65)	‐	‐	‐
Branch flexibility	‐	Low (57)	Intermediate (63)	High (20)	‐	‐	‐
Trunk color	‐	Light brown (18)	Brown (30)	Dark brown (36)	Brown‐black (15)	Brown‐gray (24)	Gray‐black (17)
Trunk type	‐	Single‐trunk (108)	Multi‐trunk (32)	‐	‐	‐	‐
Trunk diameter	‐	Low (7)	Intermediate (88)	High (45)	‐	‐	‐
Canopy symmetry	No (45)	Yes (95)	‐	‐	‐	‐	‐
Canopy density	‐	Low (38)	Intermediate (42)	High (60)	‐	‐	‐
Tendency to produce suckers	Absent (54)	Low (69)	Intermediate (13)	High (4)	‐	‐	‐
Leaf density	‐	Low (6)	Intermediate (31)	High (103)	‐	‐	‐
Leaf shape	‐	Ovate (53)	Lanceolate (52)	Elliptical (35)	‐	‐	‐
Leaf apex shape	‐	Acute (79)	Obtuse (33)	Rounded (28)	‐	‐	‐
Leaf upper surface color	‐	Light green (3)	Green (95)	Dark green (42)	‐	‐	‐
Leaf lower surface color	‐	Light green (107)	Green (33)	‐	‐	‐	‐
Leaf margin serration	Absent (4)	Present (136)	‐	‐	‐	‐	‐
Leaf serration depth	Absent (4)	Low (50)	Intermediate (57)	High (29)	‐	‐	‐
Leaf serration shape	‐	Entire (4)	Serrate (86)	Crenate (31)	Lobate (19)	‐	‐
Thorn presence on current shoot	Absent (131)	Present (9)	‐	‐	‐	‐	‐
Ripening time	‐	Late Sep (124)	Early Oct (16)	‐	‐	‐	‐
Fruit yield	‐	Low (19)	Intermediate (61)	High (60)	‐	‐	‐
Fruit shape	‐	Oblate (20)	Cylindrical (37)	Round (5)	Ovate (63)	Oval (15)	‐
Fruit skin transparency	‐	Low (41)	Intermediate (73)	High (26)	‐	‐	‐
Fruit flesh color	‐	Light cream (12)	Cream‐yellow (24)	Cream (23)	Cream‐brown (46)	Brown (17)	Cream‐olive (18)
Fruit taste	‐	Sour‐sweet (16)	Slightly sweet (3)	Sweet (115)	Very sweet (6)	‐	‐
Fruit flesh texture	‐	Soft (102)	Crisp (38)	‐	‐	‐	‐
Stone shape	‐	Elongate (50)	Oval (56)	Ovate (13)	Round (21)	‐	‐
Stone surface	‐	Smooth (6)	Relatively smooth (15)	Coarse (119)	‐	‐	‐
Stone terminal appendix	Absent (99)	Short (22)	Large (19)	‐	‐	‐	‐

In the accessions having thorn, thorn length on annual shoot ranged from 1.57–38.84 mm. Khakdaman et al., (2007) reported the range of 20.00 to 50.00 mm for thorn length. Ghazaeian (2015) studied jujube genotypes from Golestan province in Iran and reported that thorn length ranged between 10.60–20.20 mm.

**FIGURE 1:**
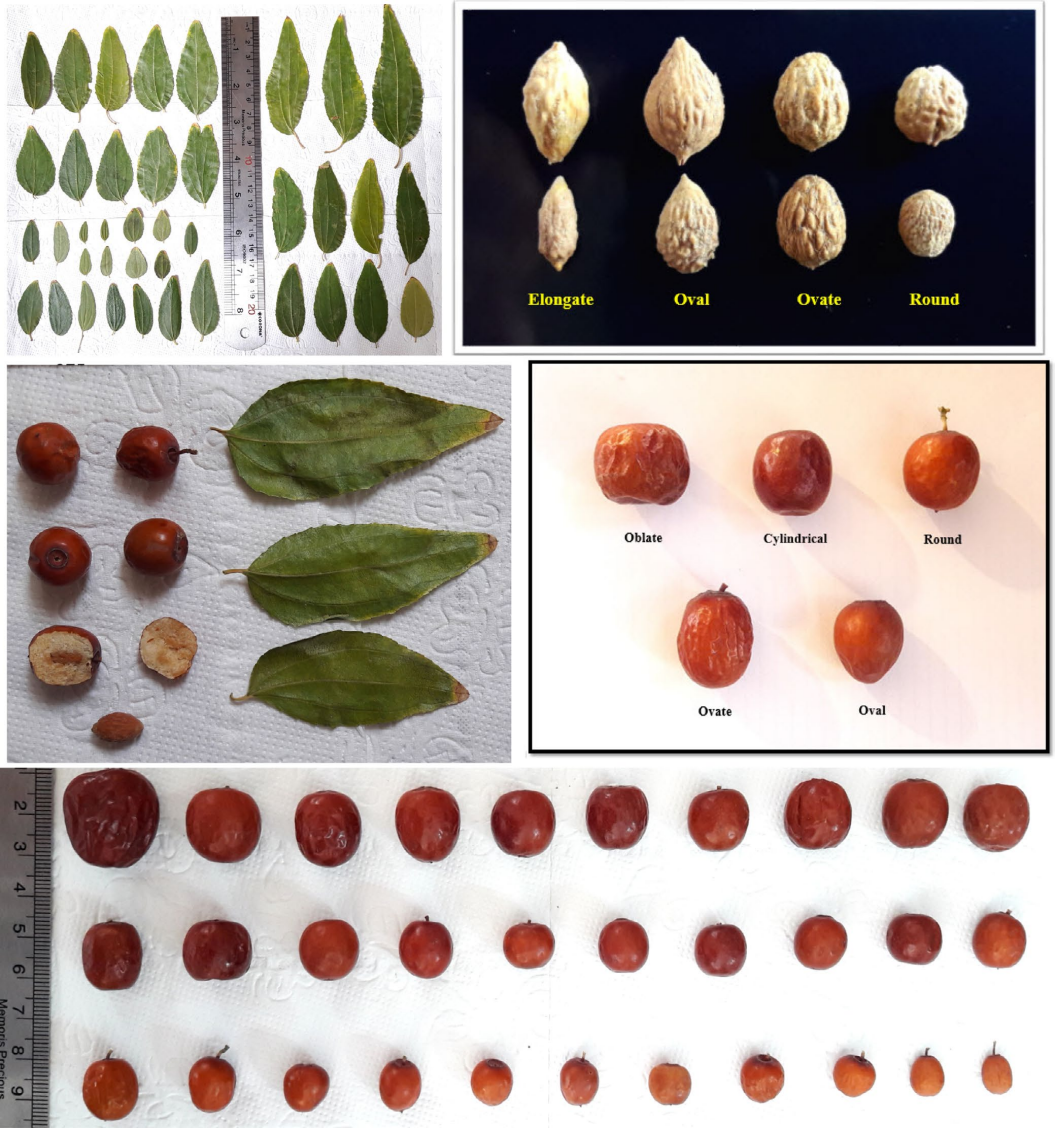
The pictures of leaves and fruits of *Z. jujuba* accessions studied

In addition, leaf apex had three shapes, including acute (79 accessions), obtuse (33), and rounded (28). Leaf margin serration was present in the majority of accessions (136) and in those accessions; leaf serration depth was low in 50, intermediate in 57, and high in 29 accessions, and also leaf serration shape was serrate in 86, crenate in 31, and lobate in 19 accessions (Figure 1). Leaf length ranged from 26.33 to 84.05 mm, and leaf width varied from 11.02 to 32.87 mm. Petiole length ranged from 1.55 to 10.60 mm, and petiole thickness varied from 0.37 to 1.39 mm (Table 2). In a similar research, the value of these traits was different between 19.00–30.00 mm and 9.00–17.50 mm, respectively (Khakdaman et al., 2007). In addition, Gao et al., (2009) showed significant variations among *Z. jujuba* genotypes from the Loesse Plateau of China in terms of leaf length and width. Ghazaeian (2015) studied jujube genotypes from Golestan province in Iran and reported the range of 25.00–56.00 mm for leaf length, 13.60–24.60 mm for leaf width, and 0.20–4.10 mm for petiole length.

Ripening time ranged from late September (124 accessions) to early October (16). Fruit yield was low in 19, intermediate in 61, and high in 60 accessions. The accessions were clustered into five groups based on fruit shape, including oblate (20 accessions), cylindrical (37), round (5), ovate (63), and oval (15) (Figure 1). Grygorieva et al., (2014) reported round, oval, apple, egg, and pear shapes in fruits of Ukrainian *Z. jujuba* genotypes.

Fruit length ranged between 9.43 and 49.47 mm, and fruit width varied between 8.38 and 30.17 mm. Fruit fresh weight ranged from 0.36 to 3.83 g with an average of 1.16, fruit dry weight varied from 0.21 to 3.04 g with an average of 0.80, and fruit flesh thickness varied from 1.24 to 8.51 mm. The range of fruit stalk length was 1.19–8.81 mm, and fruit stalk diameter was 0.35–2.98 mm. Ghazaeian (2015) studied jujube genotypes from Golestan province in Iran and reported the range of 14.60–21.30 mm for fruit length, 15.30–21.60 mm for fruit width, and 0.79–4.80 g for fruit weight. Liu et al., (2009) showed that the average fruit weight ranged from 0.14 to 6.33 g.

Fruit skin color showed large variabilities among the accessions and included maroon‐yellow (2 accessions), light maroon (10), maroon (30), dark maroon (32), light brown (4), brown (48), dark brown (8), and maroon‐crimson (6). Grygorieva et al., (2014) reported brownish‐yellow, golden‐yellow, and reddish to dark brown skin colors in Ukrainian *Z. jujuba* genotypes. Furthermore, there was high diversity among the accessions based on fruit flesh color, including light cream (12 accessions), cream‐yellow (24), cream (23), cream‐brown (46), brown (17), and cream‐olive (18) (Table 3). Grygorieva et al., (2014) reported that fruit flesh demonstrated varying consistency and different colors in Ukrainian *Z. jujuba* genotypes. Fruit taste was predominantly sweet (115), and soft fruit flesh texture was predominant (102).

Stone shape formed five groups, including elongate (50 accessions), oval (56), ovate (13), and round (21). The range of stone length and width was 6.76–15.57 mm and 3.54–9.05 mm, respectively. The range of 10.58–14.35 mm has been reported for stone length in Chinese jujube (Brindza et al., 2011). Grygorieva et al., (2014) reported the range of 12.84–28.67 mm for stone length and 5.06–9.74 mm for stone width. Stone weight ranged between 0.04 and 0.53 g with an average of 0.19. Sivakov et al., (1988) reported that the range of stone weight in six cultivars of *Z. jujuba* varied from 0.28 to 0.65 g, while Ghosh and Mathew (2002) recorded the range of 0.06–1.90 g for stone weight in *Z. jujuba*. Ghazaeian (2015) studied jujube genotypes from Golestan province in Iran and reported the range of 10.20–13.50 mm for stone length, 3.80–7.90 mm for stone width, and 0.26–1.93 g for stone weight.

### 3.2 Correlations among the measured characters

Pearson correlation analysis showed significant relationships between the characters (data not shown). Tree vigor was significantly correlated with tree height (*r* = 0.59), branching (*r* = 0.56), branch density (*r* = 0.54), trunk type (*r* = 0.19), trunk diameter (*r* = 0.43), and canopy density (*r* = 0.43). Leaf length showed significant and positive correlations with leaf width (*r* = 0.72), petiole length (*r* = 0.57), and petiole thickness (*r* = 0.52).

Fruit length was significantly and positively correlated with leaf length (*r* = 0.34), leaf width (*r* = 0.23), and fruit width (*r* = 0.71) and corresponded with the findings of Grygorieva et al., (2014). Fruit fresh weight showed significant and positive correlations with leaf length (*r* = 0.19), leaf width (*r* = 0.17), fruit length (*r* = 0.70), fruit width (*r* = 0.75), fruit stalk length (*r* = 0.19), fruit stalk diameter (*r* = 0.49), stone length (*r* = 0.57), stone width (*r* = 0.52), and stone weight (*r* = 0.73) and corresponded with the findings of Grygorieva et al., (2014). The observed correlations between traits can be used for breeding (Falconer & Mackay, 1996).

### 3.3 Principal component analysis (PCA)

The PCA is used to identify the most important features in the whole data set. It is also based on a very robust statistical model. Using PCA, different traits can be discussed in terms of components, each containing several traits (Martino et al., 2008). The PCA indicated that morphological traits were classified into 17 main components accounting for 75.44% of total variance (Table 4). The PC1 explained 12.16% of total variance and showed positive and significant correlations with fruit length, fruit width, fruit fresh weight, fruit dry weight, fruit stalk diameter, fruit flesh thickness, stone length, and stone weight that can be called fruit size component. The PC2 accounted for 7.16% of total variance with positive and significant correlations with branching, branch density, and canopy density that can be termed vegetative‐related traits component. The PC3 was called as leaf size and had positive and significant correlations with leaf length, leaf width, and petiole length, and petiole thickness and accounted for 5.95% of total variance. The above traits showed the most variation among the accessions and had the most influence on differentiating accessions.

**TABLE 4:** Eigenvalues of the principal component axes from the PCA of morphological characters in the studied *Z. jujuba* accessions

Character	Component																
	1	2	3	4	5	6	7	8	9	10	11	12	13	14	15	16	17
Tree growth habit	0.09	−0.39	0.00	−0.08	−0.10	0.15	0.38	0.28	−0.33	0.09	−0.05	0.09	−0.11	−0.13	0.07	0.39	0.25
Tree vigor	0.01	0.56	0.08	−0.02	0.25	0.57**	0.16	0.03	−0.08	0.06	−0.01	−0.02	0.02	0.05	−0.03	−0.01	0.19
Tree height	0.11	0.15	0.13	−0.03	0.03	0.81**	0.09	0.11	−0.06	0.12	−0.04	−0.04	−0.03	−0.13	0.01	0.14	0.00
Branching	0.01	0.91**	0.12	−0.01	0.05	0.06	−0.03	−0.05	−0.07	0.11	−0.01	0.07	0.11	0.01	0.05	0.00	0.02
Branch density	0.00	0.91**	0.15	0.01	0.06	0.04	0.02	−0.07	−0.07	0.05	0.01	0.04	0.07	0.03	0.03	0.04	0.08
Branch flexibility	0.27	0.01	0.01	−0.11	−0.17	−0.04	0.21	0.06	0.70**	0.02	0.06	−0.03	−0.03	0.08	0.11	0.04	0.18
Trunk color	−0.21	−0.13	−0.05	0.19	0.24	0.24	−0.02	0.12	0.45	0.03	0.26	−0.08	−0.16	−0.04	−0.09	0.14	−0.30
Trunk type	−0.07	0.02	0.10	0.29	−0.04	0.52	0.17	−0.30	−0.06	−0.02	0.12	−0.01	0.19	0.13	0.04	−0.02	−0.23
Trunk diameter	0.09	0.35	0.08	−0.01	0.19	0.47	−0.16	0.15	0.24	0.14	0.21	−0.34	−0.05	0.14	0.02	0.07	−0.04
Canopy symmetry	0.01	−0.03	0.08	0.04	0.07	0.07	0.15	0.81**	0.06	0.01	−0.01	0.00	0.07	0.10	−0.13	0.02	0.07
Canopy density	0.03	0.72**	−0.06	−0.03	0.23	0.12	0.02	0.07	0.20	−0.05	−0.20	−0.13	−0.08	−0.06	−0.05	0.08	−0.19
Tendency to produce suckers	−0.18	0.24	0.01	0.16	0.12	0.02	0.05	−0.27	0.08	−0.05	−0.08	0.09	0.02	−0.05	−0.08	0.18	−0.65**
Leaf density	−0.03	0.37	0.09	0.13	0.14	−0.04	0.10	−0.13	0.22	−0.16	−0.21	0.05	−0.06	−0.02	−0.14	0.10	0.65**
Leaf shape	0.08	0.04	−0.09	0.11	−0.03	−0.03	−0.10	−0.05	−0.02	−0.03	0.07	0.85**	0.10	0.02	−0.13	−0.01	−0.02
Leaf apex shape	0.00	0.00	−0.18	0.00	0.11	−0.02	0.16	0.10	0.02	0.01	−0.18	−0.02	−0.05	0.80**	−0.06	−0.11	0.03
Leaf length	0.17	0.16	0.85**	−0.14	0.00	0.00	0.03	0.07	−0.01	0.08	0.10	0.14	0.03	−0.19	0.05	−0.06	−0.03
Leaf width	0.14	0.10	0.77**	0.08	0.12	0.04	−0.01	0.19	−0.12	0.04	−0.02	−0.20	−0.02	−0.25	0.12	−0.06	0.08
Leaf upper surface color	0.03	0.19	0.10	0.10	0.83**	0.06	0.15	0.05	0.00	−0.07	0.01	−0.11	0.07	0.02	−0.01	0.03	0.00
Leaf lower surface color	0.15	0.12	0.15	0.05	0.88	0.09	−0.01	−0.04	−0.07	0.04	−0.01	−0.02	−0.03	0.05	0.05	0.08	−0.03
Leaf margin serration	0.06	0.02	0.10	−0.05	−0.08	0.18	0.58**	0.28	−0.06	0.05	−0.04	−0.03	−0.13	0.18	0.30	0.19	−0.13
Leaf serration depth	0.02	−0.12	0.01	0.01	0.24	0.12	0.71**	−0.12	0.27	−0.04	−0.01	0.02	−0.07	−0.07	−0.07	−0.04	−0.08
Leaf serration shape	−0.01	0.15	0.14	0.06	0.06	−0.04	0.71**	0.12	0.03	−0.04	0.04	−0.15	0.10	0.15	−0.01	−0.06	0.13
Petiole length	0.00	0.04	0.65**	−0.23	−0.01	0.06	0.29	−0.08	0.26	−0.24	0.06	0.04	0.23	0.05	0.02	−0.10	0.12
Petiole thickness	0.11	0.03	0.73**	−0.01	0.14	0.17	0.06	−0.09	0.00	−0.02	−0.13	−0.08	0.03	0.13	0.00	0.11	−0.03
Thorn presence on current shoot	−0.03	0.15	−0.20	0.25	0.48	−0.19	0.20	0.18	−0.24	−0.01	−0.17	0.10	0.17	0.27	0.22	−0.04	0.09
Thorn number on annual shoot	−0.14	0.03	−0.15	0.75**	0.02	−0.02	−0.12	−0.02	−0.15	−0.02	0.03	0.16	0.22	−0.02	0.07	0.04	0.05
Thorn length on annual shoot	−0.22	−0.02	−0.03	0.83**	0.10	0.07	0.06	0.01	0.00	0.07	−0.08	−0.04	−0.14	−0.02	−0.12	−0.07	−0.10
Thorn base thickness on annual shoot	−0.24	−0.02	−0.03	0.88**	0.09	−0.01	0.07	0.04	0.09	0.06	−0.09	−0.05	−0.13	0.07	−0.04	0.01	0.00
Ripening time	−0.11	−0.05	0.05	−0.19	−0.16	−0.07	−0.06	0.31	0.23	0.10	−0.26	0.50	−0.29	−0.23	0.16	0.00	−0.01
Fruit yield	0.05	0.13	−0.06	0.01	0.06	0.09	−0.01	0.02	0.08	−0.07	0.12	0.00	0.03	−0.09	0.08	0.73**	−0.06
Fruit length	0.86**	0.07	0.18	−0.14	0.05	0.06	−0.01	−0.07	−0.05	0.18	0.07	0.08	−0.13	0.02	−0.07	0.08	0.07
Fruit width	0.86**	0.01	0.12	−0.02	0.00	0.02	−0.02	−0.02	0.06	−0.16	0.08	−0.01	0.02	0.13	0.06	0.09	−0.11
Fruit fresh weight	0.89**	0.05	0.00	−0.15	0.09	0.03	−0.01	0.05	0.06	−0.12	−0.10	−0.02	0.14	−0.06	0.15	−0.09	0.09
Fruit dry weight	0.89**	0.06	−0.03	−0.16	0.09	0.10	−0.03	0.04	0.11	−0.08	−0.09	−0.03	0.12	−0.06	0.12	−0.07	0.03
Fruit shape	−0.17	0.03	−0.07	0.10	−0.07	−0.02	−0.03	0.21	0.06	0.64**	−0.15	0.07	0.11	−0.05	0.02	−0.01	−0.01
Fruit skin transparency	0.14	0.03	0.14	−0.05	0.08	−0.02	0.07	−0.15	0.03	0.08	0.02	−0.15	−0.01	−0.06	0.78**	0.08	0.00
Fruit stalk length	0.31	0.03	0.26	−0.23	−0.12	−0.21	−0.07	0.20	−0.19	0.19	−0.07	0.40	−0.28	0.10	−0.24	−0.07	0.04
Fruit stalk diameter	0.70**	0.08	0.33	−0.03	0.04	−0.11	0.02	0.09	−0.03	0.09	0.04	0.03	−0.24	0.08	−0.09	0.21	−0.11
Fruit skin color	−0.14	0.24	0.04	0.22	−0.03	−0.39	0.18	−0.01	−0.14	0.27	0.24	−0.09	−0.10	−0.07	−0.44	0.17	0.06
Fruit flesh color	0.08	0.10	0.12	−0.07	0.05	0.03	−0.03	0.06	0.00	0.14	−0.04	0.03	0.85**	−0.04	−0.01	0.05	−0.04
Fruit flesh thickness	0.71**	−0.08	0.23	0.01	−0.04	0.08	−0.11	−0.02	0.13	−0.08	0.08	0.04	0.03	0.19	0.12	0.14	0.09
Fruit taste	−0.15	−0.02	−0.01	0.03	0.11	0.25	−0.09	−0.04	−0.46	−0.31	0.22	−0.13	−0.23	0.08	0.03	−0.13	0.09
Fruit flesh texture	0.14	0.33	−0.10	0.09	−0.16	0.05	0.03	0.05	−0.07	−0.05	0.34	0.10	−0.14	−0.09	0.35	−0.46	0.03
Stone length	0.64**	0.00	−0.05	−0.29	−0.01	0.07	0.13	−0.22	−0.02	0.33	−0.01	0.04	−0.03	−0.29	0.02	−0.19	0.13
Stone width	0.54	−0.21	−0.14	−0.27	−0.11	−0.22	0.31	0.13	−0.14	−0.23	−0.13	0.06	0.05	−0.28	−0.07	−0.24	0.00
Stone weight	0.75**	−0.16	−0.14	−0.18	0.00	−0.08	0.21	0.13	0.00	−0.06	−0.17	−0.01	0.16	−0.30	−0.09	−0.20	0.06
Stone shape	−0.04	−0.10	−0.05	−0.06	−0.04	−0.27	0.08	0.32	−0.05	−0.61**	−0.10	0.13	−0.04	−0.12	−0.02	0.09	0.07
Stone surface	−0.03	−0.14	−0.02	−0.12	−0.04	0.01	−0.01	−0.03	0.06	−0.08	0.82**	−0.01	−0.02	−0.14	−0.02	0.08	−0.07
Stone terminal appendix	−0.16	0.18	0.11	−0.12	0.23	0.19	0.12	−0.33	−0.12	0.37	0.36	0.10	0.04	−0.04	0.22	0.00	0.19
Total	5.96	3.51	2.91	2.83	2.30	2.19	2.13	1.72	1.64	1.61	1.56	1.53	1.44	1.44	1.42	1.39	1.37
% of Variance	12.16	7.16	5.95	5.78	4.70	4.48	4.34	3.50	3.35	3.29	3.18	3.13	2.95	2.94	2.90	2.84	2.79
Cumulative %	12.16	19.32	25.27	31.05	35.74	40.22	44.56	48.06	51.42	54.71	57.89	61.02	63.96	66.91	69.81	72.65	75.44

** Eigenvalues ≥0.57 are significant at the *p* ≤.01 level.

**FIGURE 2:**
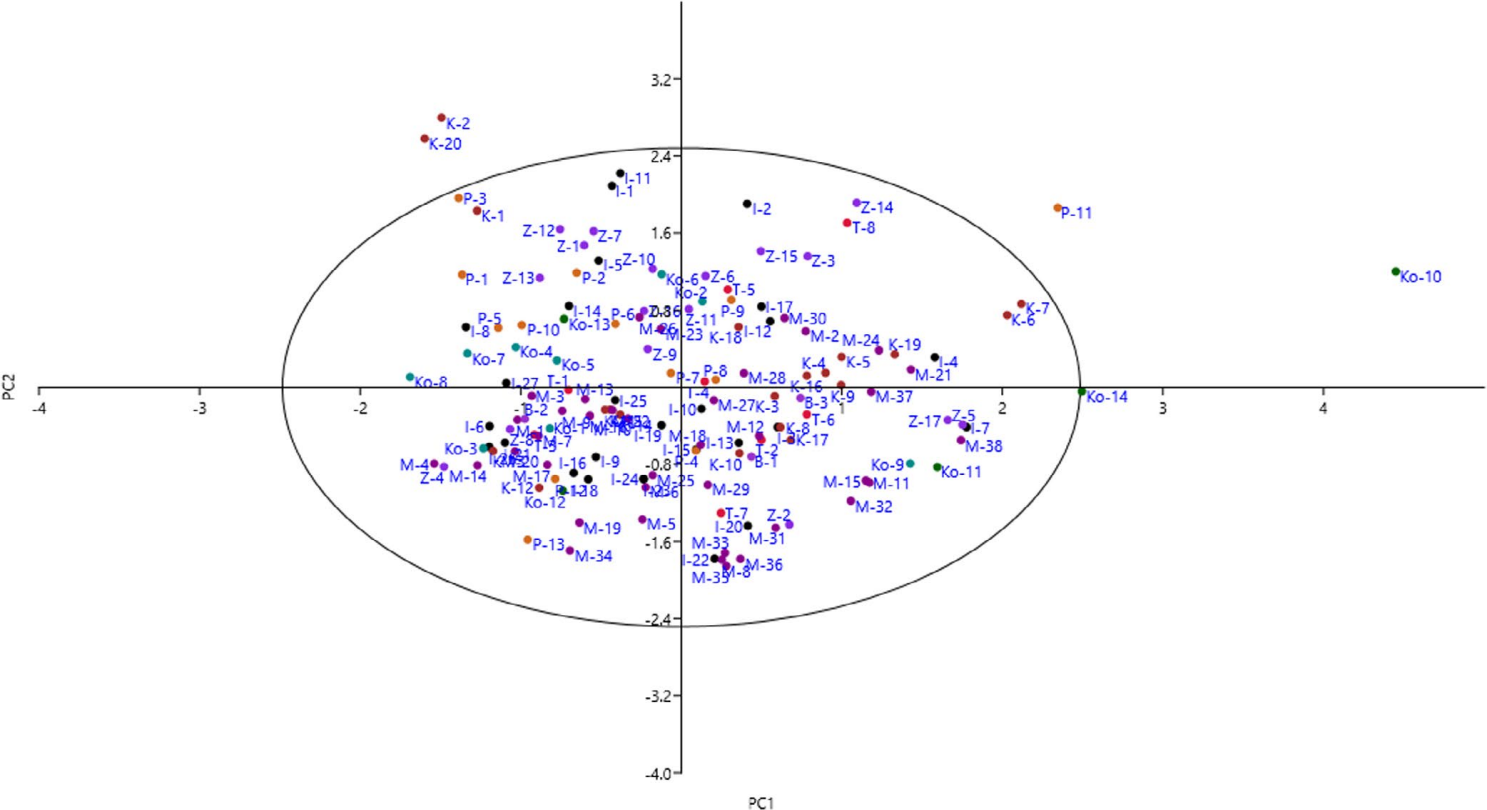
Scatter plot for the studied *Z. jujuba* accessions based on PC1/PC2. The symbols represent the accessions of each area in the plot, including Isfahan (I), Zardenjan (Z), Khatoonabad (K), Payekan (P), Tinejan (T), Koohpayeh (Ko), Khoshkrood (Kh), Mamoonyeh (M), and Barbar (B)

The scatter plot generated using the PC1/PC2 (Figure 2) showed similarity and dissimilarity among the accessions. The accumulation of accessions in one area of the plot indicated similarity between them. The accessions varied significantly in the PC1 in terms of fruit length, fruit width, fruit fresh weight, fruit dry weight, fruit stalk diameter, fruit flesh thickness, stone length, and stone weight. In the PC2, the genotypes showed a gradual increase in branching, branch density, and canopy density.

### 3.4 Cluster analysis

**FIGURE 3:**
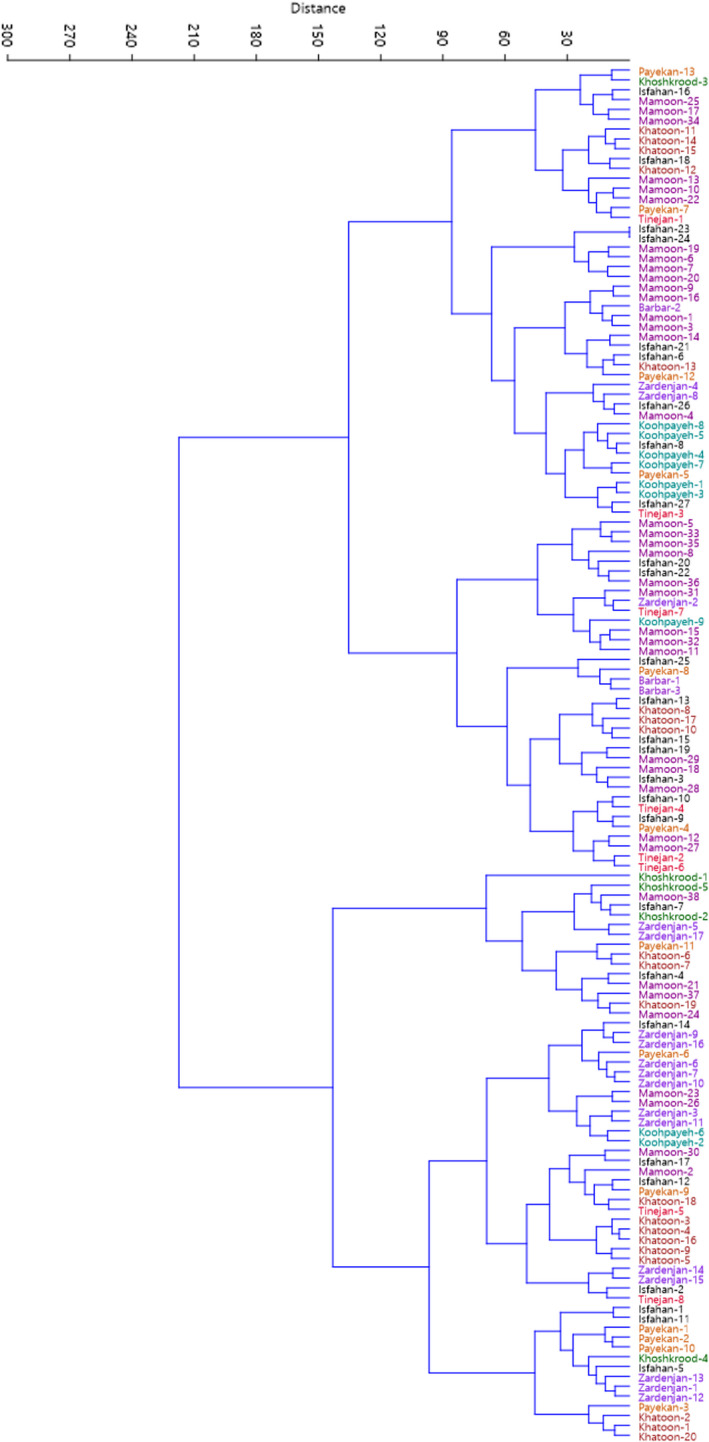
Ward cluster analysis of the studied *Z. jujuba* accessions based on morphological traits using Euclidean distances

**FIGURE 4:**
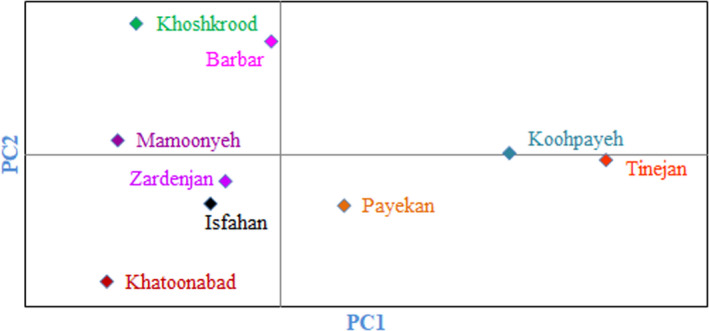
Bi‐plot for the studied populations of *Z. jujuba* based on morphological characters

Cluster analysis performed using Euclidean distance and Ward's method divided the accessions into two main clusters based on morphological traits (Figure 3). The first cluster (I) included 82 accessions, while the second cluster (II) consisted of the rest 58 accessions. Besides, results of population analysis showed that the studied populations were divided into two main groups each containing two subgroups (Figure 4). Subgroup I‐A included Koohpayeh area and subgroup I‐B consisted of Payekan and Tinejan areas both subgroups belonging to Isfahan province. Subgroup II‐A included Mamoonyeh, Khoshkrood, and Barbar areas belonging to Markazi province. Subgroup II‐B consisted of Isfahan, Zardenjan, and Khatoonabad areas belonging to Isfahan province.

There were significant variabilities among the accessions studied that could be used for conservation used breeding. Similar variability has been reported among jujube genotypes from different countries, including Spain (Almansa et al., 2016; Hernandez et al., 2016; Reche et al., 2018, 2019), Korea (Choi et al., 2011), China (Gao et al., 2011, 2012; Wang et al., 2012), Ukraine (Grygorieva et al., 2014), and Turkey (Gunduz & Saracoglu, 2014).

Jujube has high tolerance to drought and can grow in different climates. The domestication process of jujube has been based on natural reproduction and human selection (Liu & Jiang, 2008). During the long history of evolution, jujube diversity has been increased and distinct genotypes have been emerged (Liu, 2006). Awareness of leaf‐related traits can help to predict tree performance under different climates, as well as its application in breeding programs to improve varieties (Sack & Scoffoni, 2013). Analyzing leaf‐related traits in different climates can increase our knowledge of jujube adaptation strategies in response to drought stress (Ma et al., 2007).

In addition, increasing yield, fruit size, and flesh/stone ratio are some of the main goals in jujube breeding programs. The studied jujube accessions here showed high diversity in the traits related to vegetative, leaf, fruit size, fruit shape, fruit flavor, and seed, which are useful for application in breeding programs. Diversity in the traits related to fruit, flesh, and stone may reflect genetic variation of genotypes or environmental diversity in the study areas. Awareness of both factors can significantly increase the chance of selection, improvement, and genetic conservation (Wani et al., 2014). The variation observed in the same population can be due to the genetic effect (Karadeniz, 2002). In addition, self‐ and cross‐incompatibility, which is a common trait in the genus *Ziziphus*, may result in increased genetic and phenotypic variation in a population (Azam‐Ali et al., 2001). However, high differentiation may be the result of habitat fragmentation that results in the separation of populations, reducing their size, and limiting gene flow among them (Ferrazzini et al., 2008).

## 4 CONCLUSIONS

Decreased genetic diversity can make plants vulnerable to stress and may lead to the extinction of those plants, especially in harsh conditions. There was considerable diversity among the *Z. jujuba* accessions based on the recorded traits, and morphological variation including quantitative and qualitative traits appeared to be high within and between the studied populations. In addition, the observed morphological variation in the recorded traits is largely related to genetics and environment. The present findings provided essential data about the morphological traits of *Z. jujube* that can be used for the selection of superior accessions and development of the fruit processing industry. Besides, the available results can be useful in designing conservation strategies and breeding of *Z. jujube*.

## CONFLICT OF INTEREST

The authors declare no conflict of interest.

## ETHICAL STATEMENTS

Research involving Human Participants and/or Animals: None.

## Data Availability

The data that support the findings of this study are available from the corresponding author upon reasonable request.
